# Melanoma segmentation using deep learning with test-time augmentations and conditional random fields

**DOI:** 10.1038/s41598-022-07885-y

**Published:** 2022-03-10

**Authors:** Hassan Ashraf, Asim Waris, Muhammad Fazeel Ghafoor, Syed Omer Gilani, Imran Khan Niazi

**Affiliations:** 1 Department of Biomedical Engineering and Sciences, School of Mechanical and Manufacturing Engineering (SMME), National University of Sciences and Technology (NUST), Islamabad, 44000 Pakistan; 2 Department of Health Science and Technology, Aalborg University, 9220 Aalborg, Denmark; 3 Center of Chiropractic Research, New Zealand College of Chiropractic, Auckland, 1149 New Zealand; 4 Faculty of Health and Environmental Sciences, Health and Rehabilitation Research Institute, AUT University, Auckland, 0627 New Zealand

**Keywords:** Diseases, Health care

## Abstract

In a computer-aided diagnostic (CAD) system for skin lesion segmentation, variations in shape and size of the skin lesion makes the segmentation task more challenging. Lesion segmentation is an initial step in CAD schemes as it leads to low error rates in quantification of the structure, boundary, and scale of the skin lesion. Subjective clinical assessment of the skin lesion segmentation results provided by current state-of-the-art deep learning segmentation techniques does not offer the required results as per the inter-observer agreement of expert dermatologists. This study proposes a novel deep learning-based, fully automated approach to skin lesion segmentation, including sophisticated pre and postprocessing approaches. We use three deep learning models, including UNet, deep residual U-Net (ResUNet), and improved ResUNet (ResUNet++). The preprocessing phase combines morphological filters with an inpainting algorithm to eliminate unnecessary hair structures from the dermoscopic images. Finally, we used test time augmentation (TTA) and conditional random field (CRF) in the postprocessing stage to improve segmentation accuracy. The proposed method was trained and evaluated on ISIC-2016 and ISIC-2017 skin lesion datasets. It achieved an average Jaccard Index of 85.96% and 80.05% for ISIC-2016 and ISIC-2017 datasets, when trained individually. When trained on combined dataset (ISIC-2016 and ISIC-2017), the proposed method achieved an average Jaccard Index of 80.73% and 90.02% on ISIC-2017 and ISIC-2016 testing datasets. The proposed methodological framework can be used to design a fully automated computer-aided skin lesion diagnostic system due to its high scalability and robustness.

## Introduction

Skin cancer is one of the most rapidly growing causes of mortalities. World health organization reports that skin cancer causes one in three cancer cases, and it is the most common type of cancer in the U.S., with 5 million patients each year^1^. Health treatment is improved as early diagnosis of skin cancers is factored in. The first sign of pigmentation occurs as melanocytes proliferate uncontrollably. This form of skin cancer, known as melanoma, is considered a malignant tumor. Melanoma, the most lethal form, claims over 9000 lives per year globally^1^. Melanoma has a 5-year survival rate of 95% for cancer patients at advanced stages and mortality of 1.62% for those with melanoma at early stages^2^. Since early diagnosis is crucial in melanoma, early treatment and recovery are also particularly important.

As equipment and professional human resources are usually not available for each patient to be tested, an automated computer-aided diagnostic (CAD) system is needed to determine skin lesions such as melanoma, nonmelanoma, and benign. For the proper interpretation of dermoscopic images and the betterment of objectivity of the visual interpretation of dermoscopic images, well-trained and generalized CAD systems are needed. It is also possible to use CAD programs to track benign skin lesions by offering a quantitative and objective assessment to prevent their evolution to malignant lesions. Four recent experiments showed a lower diagnostic accuracy of dermatologists for the diagnosis of melanoma than the dermoscopy CAD systems^3^. Generally, a typical CAD system for skin cancer consists of four main stages: acquisition of the image, preprocessing, segmentation of skin tumor, and classification of the lesions.

The deep learning techniques have recently been extended to skin lesion segmentation. In 2016, the International Skin Imaging Collaboration (ISIC) hosted the first public benchmark competition on dermoscopic image processing to aid researchers in the field of automatic melanoma diagnosis^4^. The participants were classified using the jaccard index (JAC), with the highest participant receiving an 84.30% JAC. For the skin lesion segmentation, a multistage cascade FCN, incorporated with parallel integration (P.I.) scheme, was introduced by Bi et al. (2017)^5^. To evaluate the performance of proposed model, the authors trained the model on the ISIC-2016 training set and evaluated on ISIC-2016 and PH2 datasets. They earned average JACs of 84.64% and 83.99%, respectively, and dice coefficient indices of 91.18% and 90.66%. Yuan et al. (2017) introduced a deep FCN skin lesion segmentation methodology that used the Jaccard Distance as a loss function to resolve the imbalance between tissue-lesion pixels^6^. On the ISIC-2016 dataset, their system had the highest overall JAC of 84.70% to date.

Lin et al. (2017) compared two skin lesion segmentation methods, C-means clustering and U-Net-based histogram equalization, using the ISIC-2017 dataset to assess this work^7^. The dice coefficient index of their clustering strategy was 61% lower than the 77% obtained by the U-Net system. Goyal et al. (2017) used FCN to construct a multi-class semantic segmentation that segmented three distinct forms of skin lesions from the ISIC-2017 dataset^8^. Their procedure produced dice indices of 78.5%, 65.3%, and 55.7%, respectively for benign, melanoma, and seborrheic keratosis lesions. Yuan et al. (2017) published a study that used deep convolutional-deconvolutional neural network to segment skin lesions (CDNN), and by using the ISIC-2017 dataset, they trained their model with different colors spaces of dermoscopy images^9^. Their system came first in the ISIC 2017 Challenge, with a Jaccard index of 76.5%. Kashan et al. (2020) introduced a fully automatic skin lesion segmentation by merging U-Net and Res-Net and validated their model on the ISIC-2017 and PH2 datasets, with average JAC of 77.20% and 85.40%, respectively^10^. Nathan and Kansal suggested Lesion-Net, a skin lesion segmentation technique based on U-Net^11^. Instead of using a single loss function, their approach uses a mean of cross-entropy and dice loss. On the ISIC-2016, ISIC-2017, and PH2 datasets, the Lesion-Net obtained overall JAC of 86.47%, 78.28%, and 83.84%, respectively.

Despite the significant progress made over last few years, the literature shows that most studies focus on the quantitative evaluation of the proposed techniques. Few studies have tested proposed methodology in terms of qualitative assessment, and none of the studies have ever highlighted the clinical relevance of proposed methodology^12^. According to the inter-observer agreement by expert dermatologists, the JAC of skin lesion segmentation should be greater than 78.6%; otherwise, the reliability of suggested methodology is doubtful and lacks clinical relevance^13^. According to other research, a JAC of 80% or higher in skin lesion segmentation tasks guarantees that the standard of segmentation is appropriate for subjective clinical assessment^14^. On the other hand, most studies either do not mention JAC or report a JAC index that is below inter-observer agreement.

We suggest a novel deep learning-based, fully automatic approach for skin lesion segmentation, including sophisticated pre and postprocessing approaches. We focus on a successful training approach to manage dermoscopic images under different retrieval environments rather than focusing entirely on deep learning network architecture, making the proposed technique highly scalable. Preprocessing, model training, and postprocessing are the three steps of proposed system. During the preprocessing phase, we combine morphological filters with an inpainting algorithm to eliminate unnecessary hair structures from the dermoscopic images. For the first time, we investigate how to utilize the discriminative capacity of UNet-based three separate semantic segmentation deep neural network architectures: UNet^15^, deep residual U-Net (ResUNet)^16^, and improved ResUNet (ResUNet++)^17^ at the model training level. Finally, we use test time augmentation (TTA) and conditional random field (CRF) in the postprocessing stage to improve segmentation accuracy.

Furthermore, to address the inherent problem of biasness in semantic segmentation due to unbalanced pixel distribution, we investigate the effect of different loss functions to search for a suitable loss function that minimizes the biasness against the background of the input image. We carefully review our proposed system with the ISIC-2016, and ISIC-2017 datasets to ensure its efficacy and generalizability. The proposed technique outperforms state-of-the-art approaches in terms of qualitative and quantitative segmentation efficiency, and it can be easily extended to other complicated medical image segmentation challenges.

## Fully convolutional neural networks

**Figure 1:**
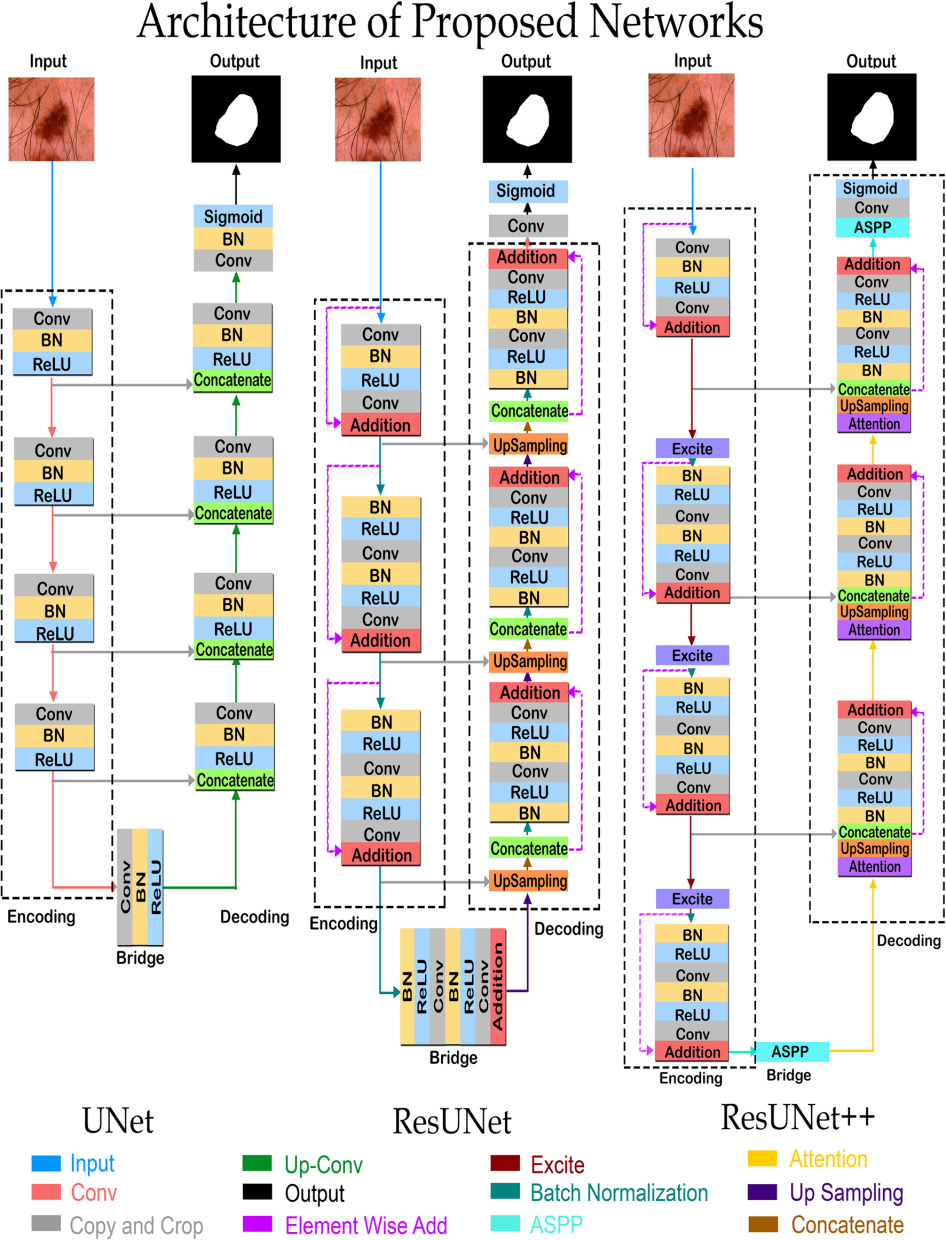
The architectures of utilized UNet, ResUNet and ResUNet++ models. All three architectures have encoder, bridge, and decoder blocks. In all these blocks 3 $\times$ 3 convolutional layers has been used except in output block, where a 1 $\times$ 1 convolutional layer followed by a sigmoid function is used to convert segmentation map into the predicted mask. Combinedly, 21, 15 and 41 convolutional layers has been used in UNet, ResUNet and ResUNet++ architectures, respectively.

### U-Net

UNet is an FCN that adds network layers to a traditional contracting network, replacing pooling operators with upsampling operators to improve the resolution of predicted image^18^. A subsequent convolution layer assembles a more specific output for localization and backward propagation by concatenating high-resolution features from upsampled output and contraction path. The network can spread context information to higher resolution layers with upsampling components, resulting in a u-shaped architecture. Owing to the absence of fully connected layers, the network uses an overlap-tile technique to conduct smooth image segmentation. The utilized architecture of the UNet model has nine blocks and 21 convolutional layers as illustrated in Fig. 1. For the smooth tiling of the output of segmentation map the size of the input tile should be integer times of 2$^n$, where n is the number of pooling layers, so that 2 $\times$ 2 max pooling operations can be used.

### Deep residual U-Net (ResUNet)

**Figure 2:**
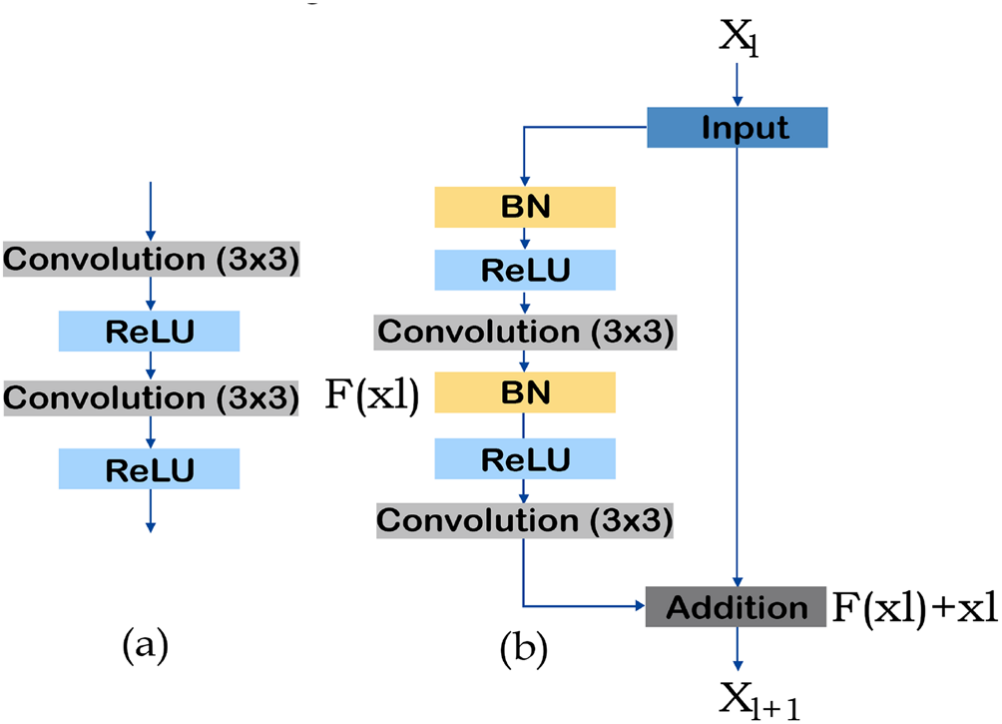
The building blocks of neural network. (**a**) shows a plain neural unit used in UNet, whereas (**b**) shows a residual unit used in ResUNet and ResUNet++ architectures.

ResUNet is an extension of UNet that incorporates the advantages of U-Net and the residual neural networks and falls in the category of semantic segmentation neural network^16^. A collection of stacked residual units make up the residual neural network. The discrepancy between a simple and a residual unit is illustrated in Fig. 2. The convolutional layers with rectified linear unit (ReLU) activation functions and batch normalization make several standard configurations in a single residual unit. The residual unit makes network training easier since the skip-links allow information to propagate without degradation.

Comprised of encoding, bridge, and decoding blocks, the utilized ResUNet architecture has seven sublevels. The input image is first transformed into dense representations in the encoding block. The final section returns the representations to pixel-by-pixel categorization. The middle section acts as a link between the decoding and encoding blocks. Residual units of all three sections consist of an identity mapping block and two 3 $\times$ 3 convolution blocks. Each convolutional block comprises a convolutional layer, batch normalization layer, and RELU activation function.

### ResUNet++

Squeeze and excitation blocks, attention block, residual block, and atrous spatial pyramidal pooling (ASPP) are introduced in the architecture of ResUNet++^17^. The residual block propagates dense feature maps to the next unit to address degradation problem of each encoder block while decreasing the computation cost. Three encoders, three decoders, and an ASPP block make up a single stem block in the proposed ResUNet++ architecture. Figure 1 illustrates the proposed ResUNet++ architecture in block diagram form.

Each convolution block comprises a single convolutional layer, batch normalization layer, and an activation function (RELU). The spatial dimension of the generated feature maps is reduced to half using a strided convolutional layer in the first convolutional layer of encoder block. The input of squeeze and excitation block is the output of previous encoder unit. ASPP expands the field of view of each filter by acting as a bridge. Nearest neighbor sampling is used for upsampling the low-level feature maps, which are then concatenated with the corresponding feature maps from the encoder path. Finally, to generate the final segmentation map, the output of decoder block passes through the ASPP block, and a single convolutional layer along with a sigmoid activation function is utilized.

## Methods

### Datasets

We use two freely accessible dermoscopy image datasets in this analysis. The ISIC-16 dataset includes 379 8-bit RGB testing images and 900 8-bit RGB training dermoscopic images with corresponding annotated masks^13^. The ISIC-2017 dataset includes 2000 training images and annotated ground masks, 150 validation images and ground masks, and 600 testing images^19^. Both datasets have images of sizes ranging from 542 $\times$ 718 to 2848 $\times$ 4288 pixels.

**Figure 3:**
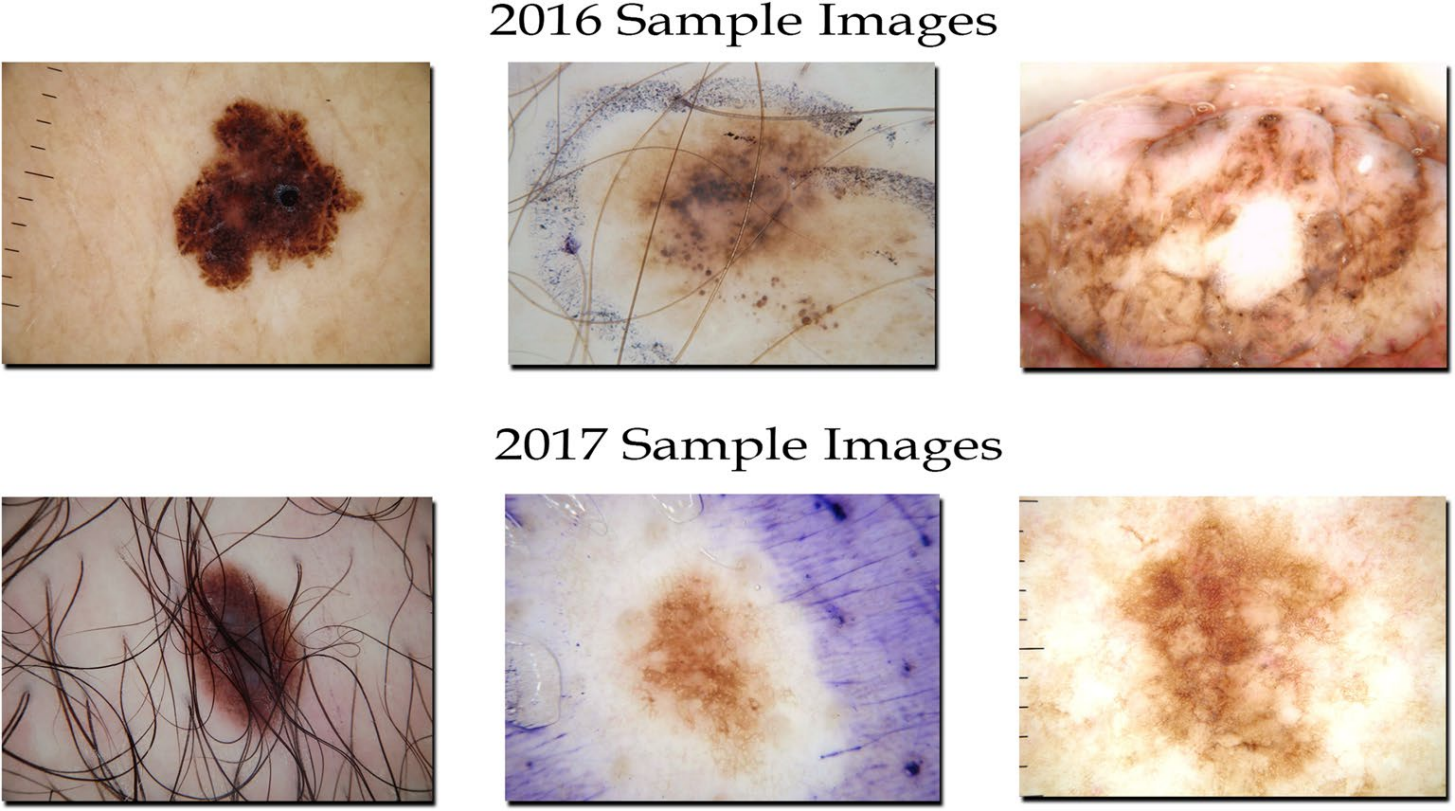
Some example dermoscopic images from ISIC-2016 and ISIC-2017 datasets. The images of both datasets vary in characteristic making the segmentation process more challenging.

Instead of training the model only on individual datasets, we also combine both datasets by combining the corresponding training images from ISIC-2016 and ISIC-2017 datasets and train our proposed frameworks on individual and combined datasets. To evaluate the efficacy of proposed model, we test the trained models on combined testing set and individual testing images of ISIC-2016 and ISIC-2017. Some of the example images from both datasets are shown in Fig. 3.

Upon exploratory analysis, we find that most of the images in both datasets are replicating; by removing the duplicate images, the newly merged dataset contains 2150 training images and 979 testing images.

### Preprocessing

All the images are resized and rescaled to 256 $\times$ 256 pixels using nearest-neighbor interpolation to resolve individual differences in dermoscopic image sizes. Inpainting algorithm and morphological filters have been used to eliminate these unwanted structures. Although inpainting in computer vision is used to retrieve the lost parts of images, we have extended the application of inpainting algorithm by utilizing it to remove hair structures from dermoscopic images. The algorithm preserves the dermoscopic appearance by replacing the parts of image with hair structures with the neighboring pixels, generating a clean dermoscopic image. The original RGB dermoscopic image is first converted into grayscale image, then morphological filter, named as black top-hat, is applied to the resulting grayscale image. If *X* is the original image, and *Y* represents the closing of *X*, then the utilized morphological filter $B_{TH}$, can be obtained as:

1 $$\begin{aligned} B_{TH}=(X\cdot Y)-X. \end{aligned}$$

**Figure 4:**
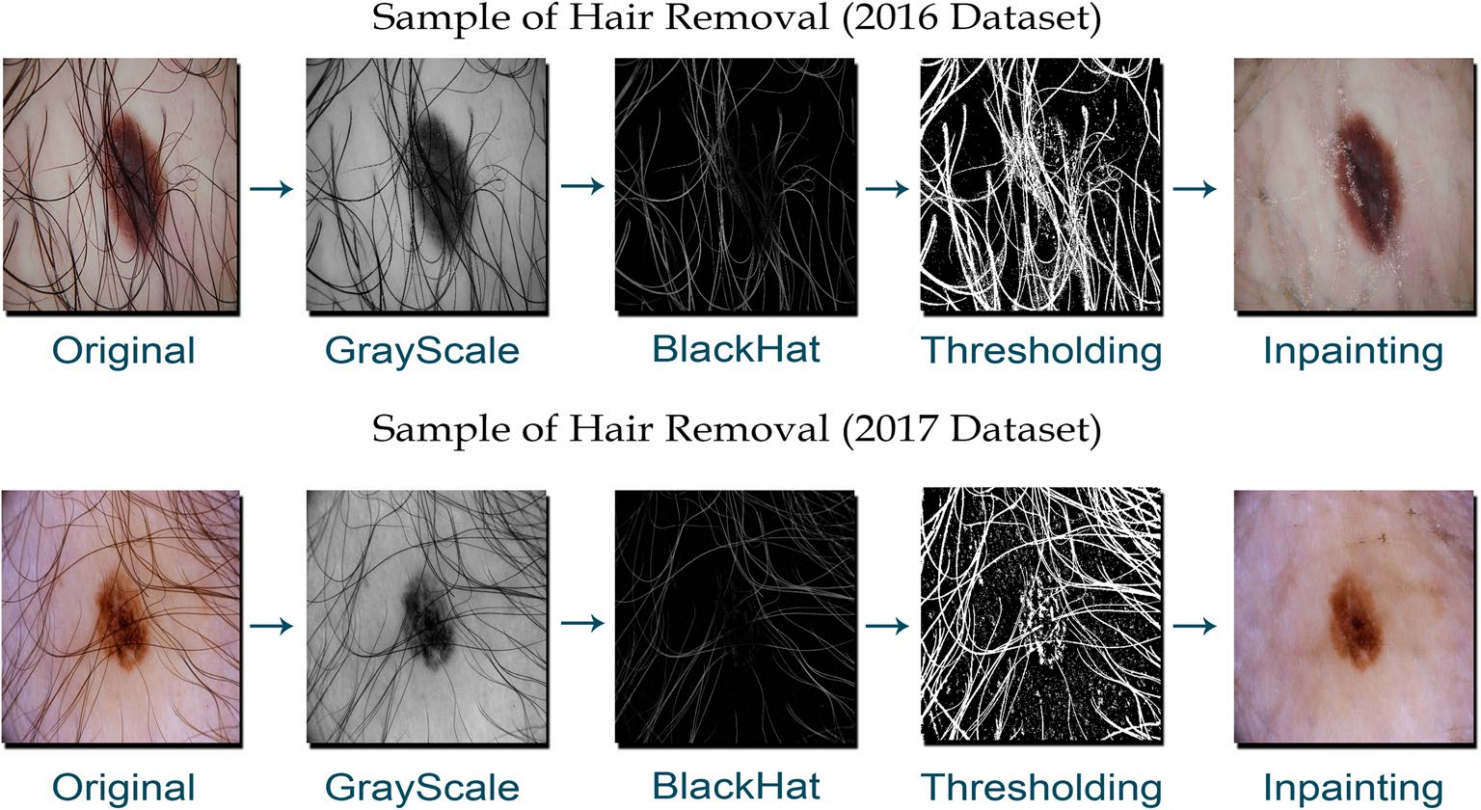
The qualitative results of the proposed hair removing technique in the preprocessing stage. The raw RGB dermoscopic image is first converted to grayscale, then a black top-hat filter followed by a thresholding operation is applied. Finally, by utilizing inpainting algorithm the hair structures are removed while preserving the shape of the image.

The morphological closing procedure covers the tiny gaps in the image while maintaining the initial area sizes. As a result, all background regions get deleted, except for pixel values that serve as structuring components. A thresholding operation is applied to the output of utilized morphological filter to generate a binary mask of undesired structures present on the dermoscopic image. Following the development of the binary mask of hair structures, an inpainting algorithm is used to extract and restore the clean skin lesion image, as shown in Fig. 4.

### Image augmentation

By increasing the training dataset with image augmentation techniques, various image transformations mitigate the overfitting problem. The robustness of the proposed model has been increased by incorporating a wide range of image acquisition conditions. Various pixel and spatial level image transformations are performed to increase the number of training images. Table 1 presents the details of the pixel and spatial level image augmentation techniques used in this study.

**Table 1:** Different type of pixel and spatial level image transformations used in this study.

Augmentation type	Augmentation
Pixel level	Motion blur
	Random brightness
	Random contrast
	Random gamma
	Hue saturation value
	RGB shift
	Grayscale
Spatial level	Random 90 rotation
	Vertical flip
	Horizontal flip

### Model training

Three fully convolutional semantic segmentation neural network architectures named as UNet, ResUNet, ResUNet++ are used to design a segmentation framework. Encoder, bridge, and decoder are three operating units of the three architectures. A translational invariant convolutional network, with the aid of convolution operation, generates the feature map F and comprises bias applied to each feature map, an activation function, kernels of the filter, and convolution operator. After each convolutional layer, a nonlinear activation process using the RELU activation function is implemented to introduce nonlinearity in the proposed network. The RELU function also helps the network resolve the degrading gradient issue and make the network more computationally effective. At the end of each proposed network, a sigmoid activation function is used to generate a final segmentation map.

An optimizer modifies the different model parameters which decreases the loss with much less effort. On all testing and validation datasets, the adaptive moment estimation (ADAM) optimizer makes quick progress in reducing the loss function and reliably converges faster than the traditional stochastic gradient descent momentum (SGDM). Besides, research has shown that the validation curve of ADAM optimizer is very smooth for segmentation tasks compared to other optimizers, making it more stable for segmentation tasks^14^. As a result, we use the ADAM optimizer for all of our experiments in this research.

The tradeoff between the accuracy and robustness of the model and the time it takes to learn the model parameters depends on the chosen learning rate. Therefore, finding an optimal learning rate in deep learning models is challenging. We utilize a cosine annealing-based stochastic gradient descent with restarts (SGDR) scheduler to tackle the difficult task of determining the optimal learning rate. Throughout the training phase, the SGDR scheduler steadily lowers the learning rate in predefined stages. The learning rate is initially set at $1e^{-2}$ and eventually decreases to $1e^{-4}$, with a 10% reduction after each loop. In each cycle, the original number of epochs is set to 5, and the number of epochs is restarted by a scaling factor of 1.5 after each process is finished. With an early stop criterion, the cumulative number of epochs is set at 300.

The loss function compares the class projections to the one-hot encoded target vector for each pixel. The total loss of each pixel for N images is reduced during the training stage by measuring the loss function between the actual mask (X) and the projected mask (Y) corresponding to the number of classes (c). The loss function of skin lesion segmentation task should be similar to the assessment criterion^5^. We use various loss functions to find the best suited loss function for the skin lesion segmentation task. Different loss functions used in this study are listed in Table 2.

**Table 2:** Different type of loss functions with description used in this study.

Loss function	Description
Binary cross-entropy	$BCE(X,Y)= -(X \cdot \log {(Y)}+(1-X) \cdot \log (1-Y))$
Dice loss	$DL(X,Y)=\sum _{i=1}^{c}{1- \frac{\sum _{i=1}^{N}XY+1}{\sum _{i=1}^{N}{X+Y}+1}}$
Binary cross entropy—Dice loss	${BCE}_{DL}=\ 0.2x\left( BCE\right) +0.8x(DL)$
Focal loss	BCE is used to derive Focal Loss (F.L.), cross-entropy function can be rewritten as: $$p_{c} = {\left\{ \begin{array}{ll} -\log (p)\,, &{} X= 1\\ -\log (1-p)\,, &{} \text {Otherwise}\\ \end{array}\right. }$$ $$\hbox {CE} = {\left\{ \begin{array}{ll} p\,, &{} X= 1\\ 1-p\,, &{} \text {Otherwise}\\ \end{array}\right. }$$ $\hbox {CE(X,Y)}=(\hbox {CE})(\ p_c)=-\log (p_c)$ Furthermore, the F.L. is calculated using a modulating factor $\gamma$ which ranges from 0 to 1. $\hbox {FL}(\mathrm{p}_c)=\ -\gamma (1-p_c)\ \cdot \log (p_c)$
Focal Tversky loss	$FTL(X,Y)=\ \sum _{C}{(1-\ }\frac{XY}{XY+ \beta (1-X)Y+(1-\beta )X(1-Y)})$
Tversky loss	$TL(X,Y)=\ 1-\frac{1+XY}{1+XY+\ \beta (1-X)Y+(1-\beta )X(1-Y)}$ Here, $\beta =1/2$ is utilized to adjust false positives and false negatives rates.

### Postprocessing

#### Test-time augmentation

Test-time augmentation (TTA) is a data augmentation strategy that enlarges the dataset by integrating transformations from the initial dataset and is widely utilized throughout deep learning algorithm training^20^. We can, however, use it during testing to achieve greater robustness and better precision. To produce a “smoothed” prediction, TTA pools predictions from multiple transformed copies of a given test input. The goal of TTA is to make subjective adjustments to the test images. Instead of testing the trained model with the standard “clean” images from the testing dataset just once, we test the augmented tested images repeatedly. Then we take the average of predictions of each image and use it as our final prediction.

#### Conditional random fields

Conditional random field (CRF) is a graphical model and a class of discriminative models that are ideal for prediction tasks in which spatial information or the state of the neighbor influences the present prediction^21,22^. It has been reported that FCN generates a very coarse image segmentation result^21^. To address this issue, we utilize CRF to fine-tune the rough image segmentation results depending on the label at each point and the labels and locations of nearby positions.

Primary advantage of CRF is the ability to add contextual, non-independent information features. It can develop a direct relationship between the input and output images while considering “neighboring” samples for better prediction. We use CRF by first specifying the necessary feature functions, setting the weights to random values, and then recursively introducing gradient descent till the boundary conditions converge.

### Evaluation metrics

We evaluate the predicted labels into false negatives (FN), true negatives (TN), false positives (FP), and true positives (TP). These primitives are readily used in evaluation metrics to measure the efficiency of trained model. Multiple evaluation metrics such as specificity or precision (PRE), sensitivity or recall (REC), jaccard index (JAC), and dice coefficient (DC) are used to report the efficacy of the trained models on test set. Precision measures the percentage of pixels that belong to the lesion region that are correctly segmented. Recall calculates the percentage of pixels that do not belong to the lesion area and are incorrectly segmented. The rate of Intersection over Union (IoU) of the expected and real masks is the jaccard index. Table 3 summarizes the various performance metrics used in this study.

**Table 3:** Performance evaluation metrics used to evaluate the performance of proposed skin lesion segmentation framework.

Performance metric	Description
Precision	$PRE=\ \frac{TP}{TP+FP}$
Recall	$REC=\ \frac{TP}{TP+FN}$
Jaccard index	$JAC=\ \frac{TP}{TP+FN+FP}$
Dice coefficient	$DC=\ \frac{2\ .\ TP}{(2\ \cdot \ TP)+FN+FP}$

On the other hand, the dice coefficient is the percentage of resemblance between the real and expected masks. These efficiency measurements with higher values mean that the segmentation performance of network is more outstanding. Although all these performance assessment metrics indicate the ability and correctness of semantic segmentation model in predicting masks, inter-observability of dermatologists suggests assessing network performance using the Jaccard Index^13^.

## Results

**Figure 5:**
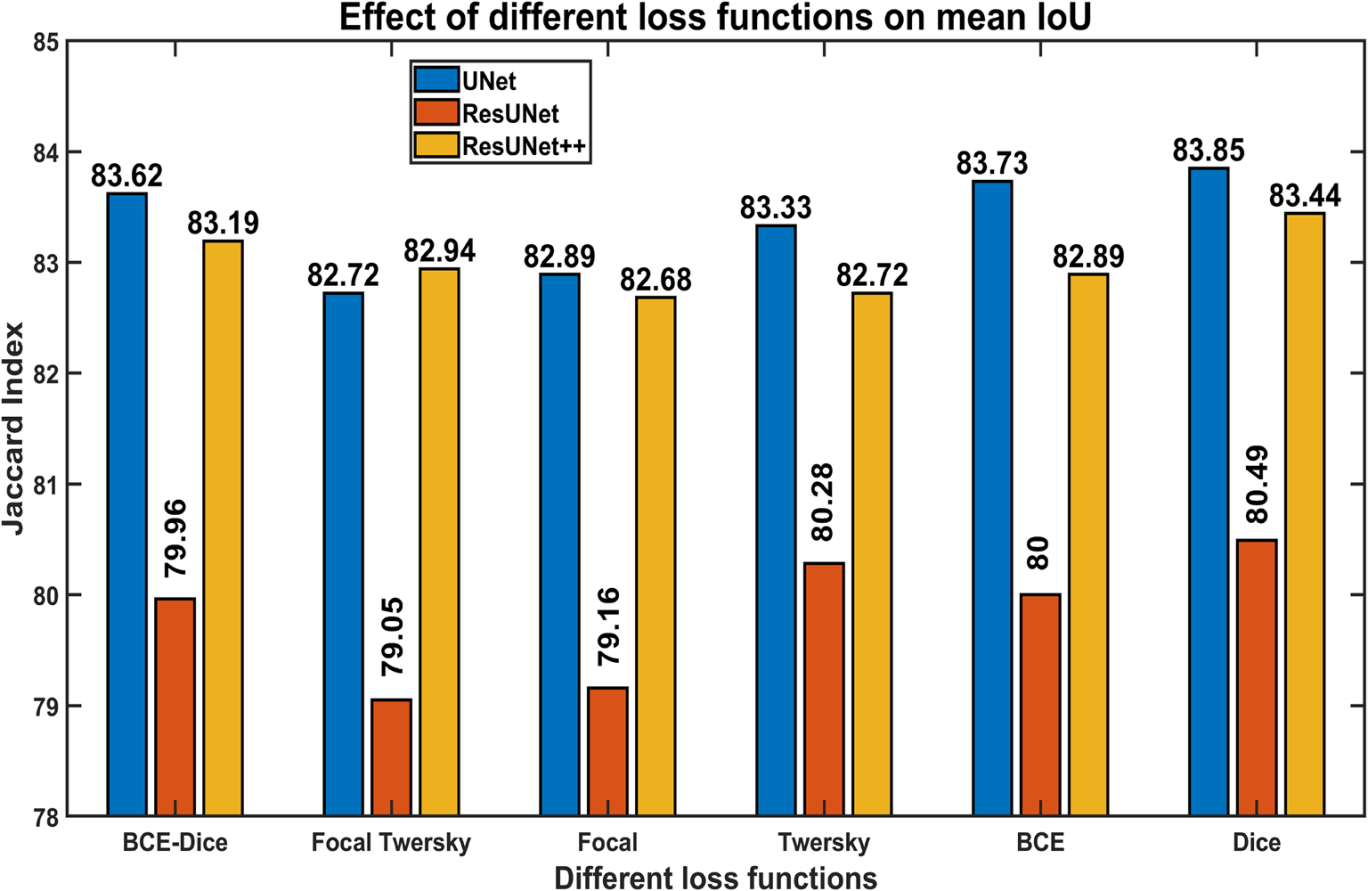
Effect of different loss functions on the performance of proposed semantic segmentation techniques. In all conducted experiments, for all three neural networks, Dice Loss achieved the highest Jaccard Index.

### Optimal configurations

Some experiments were conducted to investigate the best possible combination of parameters that affect the performance of proposed model. The effect of different augmentations, image size, loss function, and batch size has been investigated in this study and only combined (ISIC-2016 and ISIC-2017) dataset was used in these experiments for training and testing of the models.

#### Augmentations

As mentioned earlier, for the robustness of the proposed skin lesion segmentation system and to avoid overfitting, different transformations of the training images were generated. We first trained the models without any augmentation and then with varying number of image transformations. The combinations were made based on prior knowledge found in the literature^12^. In the first experiment, the model was trained on the original dataset, and no augmentations were applied. In the second experiment, three geometric augmentations (rotation, vertical and horizontal flip) were used on the training dataset. In the third experiment, seven pixel and spatial transformations (rotation, vertical and horizontal flip, hue saturation value, RGB shift, random brightness, random contrast) were applied. In the fourth experiment, all the mentioned augmentations (10 augmentations) were used. The results of all the experiments are listed in Table 4.

**Table 4:** Effect of different augmentation schemes employed in this study. The models were first trained and tested without using train-time augmentations, in next experiments different combinations were used to assess the effect of image augmentations on the performance of proposed technique.

Augmentations	Model	Jaccard index
No augmentations	UNet	82.34
	ResUNet	79.22
	ResUNet++	82.87
Rotation, vertical, and horizontal flip	UNet	82.25
	ResUNet	79.28
	ResUNet++	83.01
Rotation, vertical,horizontal flip, hue saturation value, RGB shift random brightness, and random contrast	UNet	83.39
	ResUNet	80.35
	ResUNet++	83.44
Rotation, vertical,horizontal flip, grayscale hue saturation value, RGB shift, random brightness random contrast, motion blur, and random gamma	UNet	82.72
	ResUNet	79.81
	ResUNet++	83.10

From the Table 4, it can be inferred that including the different image transformations increases the performance of proposed system. However, the highest Jaccard Index resulted from combination 3 of image augmentations, in which we used seven different image transformations. Increasing the number of augmentations to ten does not further improve the performance of proposed framework.

#### Image size

The performance of the proposed methodology for skin lesion segmentation was assessed for four different image sizes, i.e., 128 $\times$ 128, 192 $\times$ 192, 256 $\times$ 256, and 512 $\times$ 512. A larger image size yields better performance results, but it increases the computational requirements of the model; on the other hand, the smaller image size, due to loss of information, results in lower accuracy and requires less computational resources. The tradeoff between the accuracy of the system and its computation requirement suggests investigating the optimum image size for accurate skin lesion segmentation tasks. The effect of different image sizes on the performance of proposed model is illustrated in Table 5. It can be observed that increasing the image size increases the performance of the skin lesion segmentation system. The best performance resulted when the image size of the training data was set to 512 $\times$ 512 followed by image size of 256 $\times$ 256. However, due to limited computational resources, we opted for an image size of 256 $\times$ 256.

**Table 5:** The effect of different input image sizes on the performance of proposed methodology.

Method	Model	Jaccard index
128	UNet	80.35
	ResUNet	77.10
	ResUNet++	81.33
192	UNet	82.60
	ResUNet	79.56
	ResUNet++	82.49
256	UNet	83.39
	ResUNet	80.35
	ResUNet++	83.21
512	UNet	83.12
	ResUNet	80.40
	ResUNet++	83.44

#### Loss function

For semantic segmentation tasks, usually binary cross-entropy loss function is used. As in dermoscopic skin lesion images, the tumor constitutes only a tiny portion of the entire image, leading the model towards biasness for the background of dermoscopic image. Therefore, we evaluated various loss functions to find the optimum loss function for pixel-wise skin lesion segmentation tasks to address the issue. Six types of widely used, as mentioned earlier, loss functions are thus utilized in this study. The effect of different loss functions on skin lesion segmentation performance is presented in Fig. 5.

#### Batch size

Although the batch size in semantic segmentation tasks primarily affects the learning rate at which the model learns and the time it takes to converge. However, studies have indicated that smaller batch sizes take a smaller number of iterations to converge and increase the robustness and accuracy of the trained model^21^. Thus, to find the suitable batch size for the skin lesion segmentation task, we investigated our proposed methodology with a batch size of 16, 32, and 64. The effect of the batch size on the performance of the model is illustrated in Table 6. The performance of the proposed methodology degrades as the batch size is increased.

**Table 6:** Effect of batch size on the performance of proposed skin lesion segmentation system.

Method	Model	Jaccard index
16	UNet	84.11
	ResUNet	80.75
	ResUNet++	83.44
32	UNet	83.39
	ResUNet	80.35
	ResUNet++	82.67
64	UNet	82.32
	ResUNet	79.03
	ResUNet++	82.11

### Preprocessing

As the semantic segmentation models learn from the pixel values of input images, any unwanted and unnecessary information embedded in the images can drastically affect the performance of the model. Dermoscopic images in clinical settings have some unwanted hair on the images that affect the accuracy of the trained model. Morphological filters with an inpainting algorithm were used to remove these hair structures from input images. The proposed models were trained on raw and clean dermoscopic images, and the effect of proposed preprocessing technique on the accuracy of the trained models is presented in Table 7.

**Table 7:** Effect of preprocessing on the performance of proposed skin lesion segmentation system.

Method	Model	Jaccard index
With preprocessing	UNet	82.84
	ResUNet	79.34
	ResUNet++	82.45
Without preprocessing	UNet	81.39
	ResUNet	79.15
	ResUNet++	81.88

### Postprocessing

**Figure 6:**
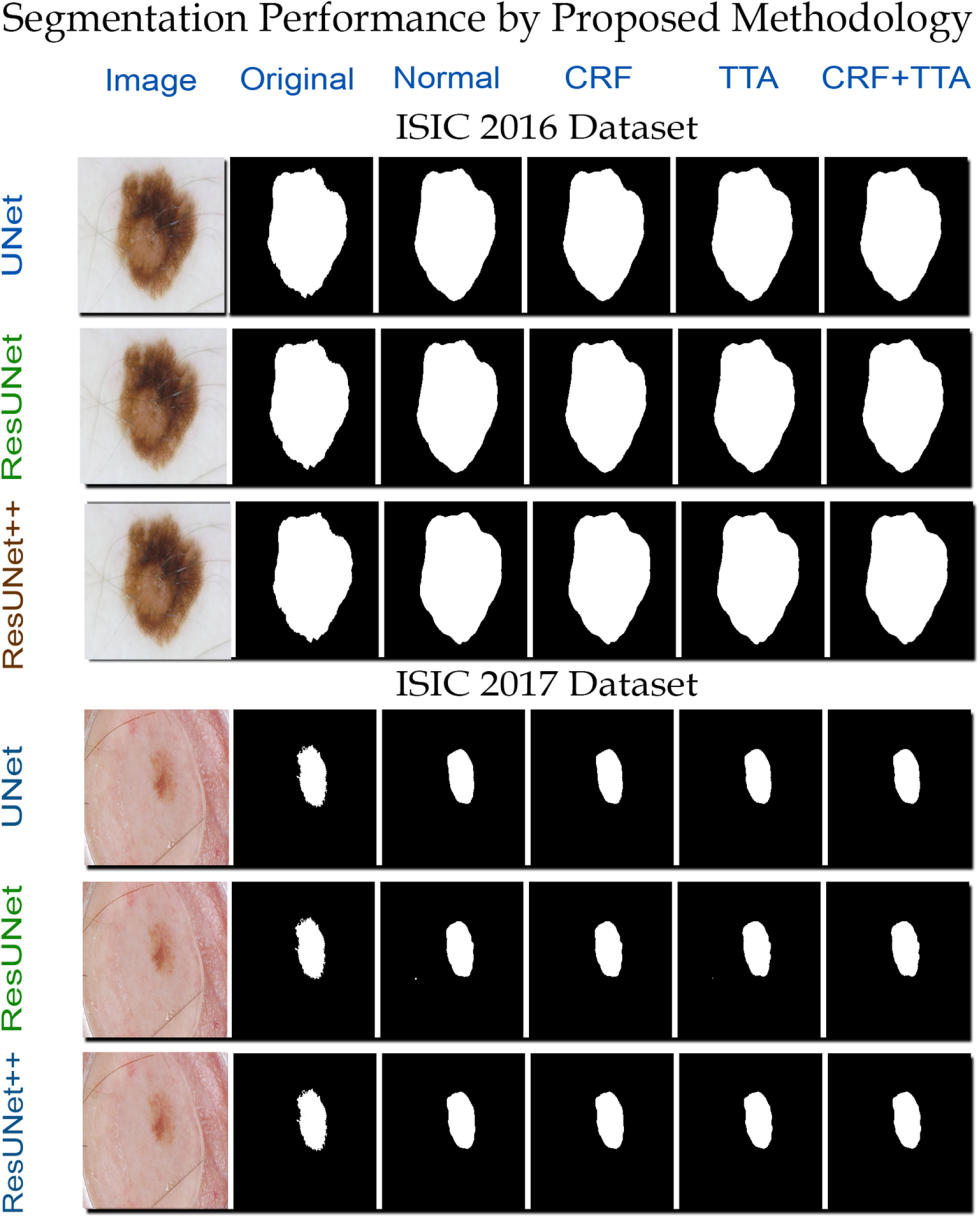
Qualitative performance of proposed skin lesion segmentation technique on ISIC-2016 and ISIC-2017 datasets. The first column shows the original testing images, the actual annotated masks are shown in column 2. The third column shows the predicted mask without using any postprocessing technique. In fourth and fifth column the predicted masks refined by CRF and TTA are shown. The last column shows the predicted mask refined by using both CRF and TTA.

The course segmentation of desired objects is the intrinsic issue in semantic segmentation frameworks. To fine-tune the predicted masks of skin tumors and increase the accuracy of the model, we utilized TTA and CRF in the postprocessing step. The resultant performance of both techniques is listed in Table 8. Figure 6 also visualize the effect of TTA and CRF on the predicted mask.

**Table 8:** Effect of postprocessing technique on the performance of proposed skin lesion segmentation system.

Model	Normal	CRF	TTA	CRF-TTA
UNet	83.84	83.94	84.59	84.60
ResUNet	80.34	80.59	80.89	81.07
ResUNet++	82.45	82.44	82.43	82.44

### Quantitative analysis

The first step in this study was to identify the best possible configurations for the proposed skin lesion segmentation methodology. We conducted several experiments, and the following parameters were determined for better performance of the models.

- Batch size: 16
- Augmentation: Combination 3 (Seven augmentations)
- Loss function: Dice Loss
- Image size: 256 $$\times$$ 256

**Figure 7:**
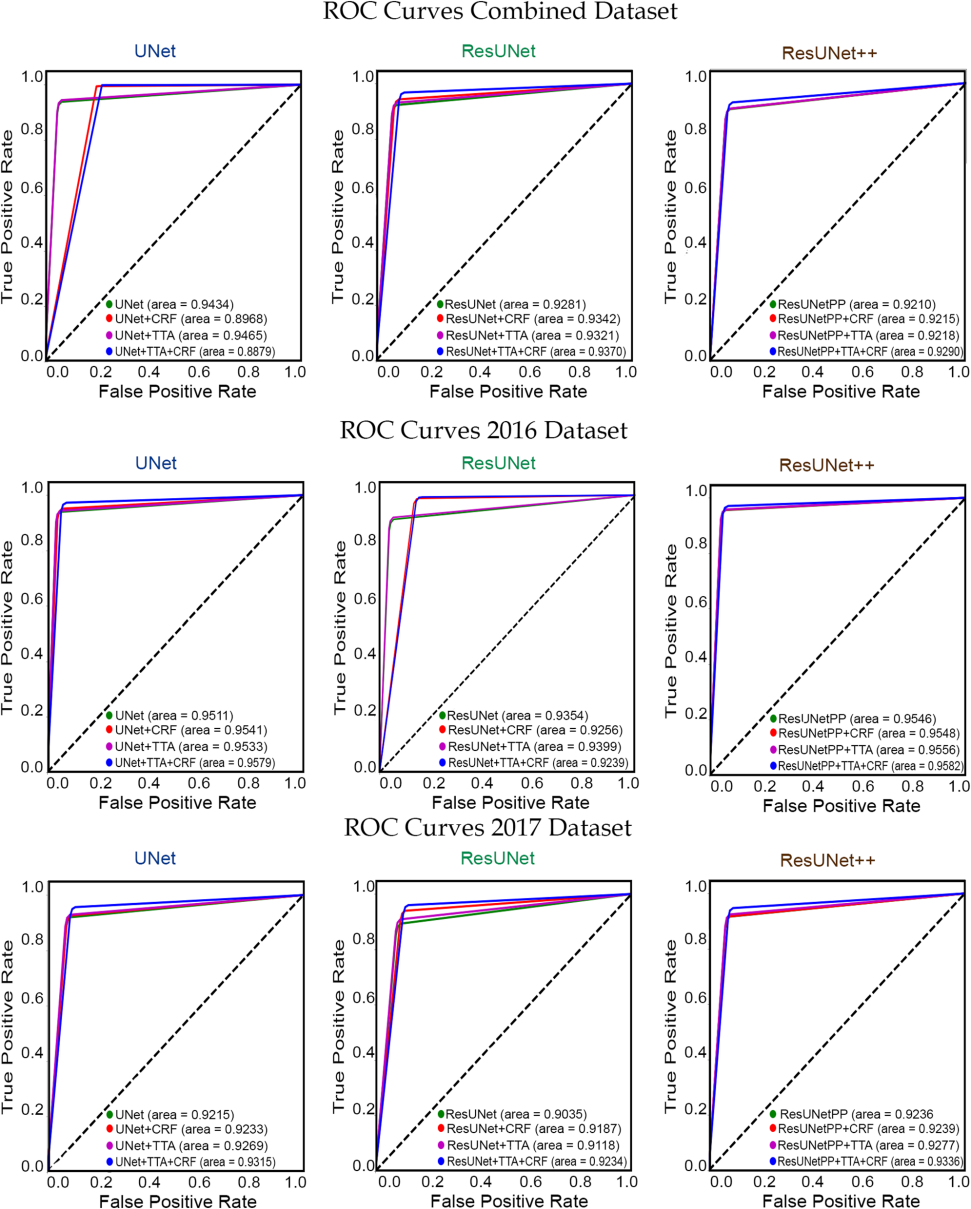
Pixel level ROCs resulted from proposed technique corresponding to all three utilized neural networks. The first row shows the area under the curves (AUCs) for the model trained and tested on combined dataset. the second and third row illustrated the AUCs for the models trained on combined data and tested on ISIC-2016 and ISIC-2017 dataset, respectively.

Based on these parameters and configuration, we propose three frameworks for the segmentations of dermoscopic images using UNet, ResUNet, ResUNet++. All the results presented in the following sections are reported from models trained on the above mentioned parameters.

#### Results of proposed methodology

This section presents the quantitative results of the proposed methodology. Based on the identified parameters, we trained three neural networks for semantic segmentation of skin lesions from the combined dataset (ISIC-2016 and ISIC-2017). The trained models were tested using the testing dataset from the combined dataset and individual testing images of ISIC-2016 and ISIC-2017. The pixel-wise segmentation performance of proposed methodology is presented in terms of Dice Coefficient, Jaccard Index, Precision, and Recall.

**Table 9:** Results of proposed methodology trained and tested on combined dataset.The results presented in this table are resulted from the models trained on combined dataset.

Method	Dice	Jaccard	Precision	Recall
**Trained and tested on combined dataset**				
UNet	87.26	84.60	89.18	86.10
ResUNet	83.59	81.07	84.61	85.06
ResUNet++	83.73	85.44	86.11	93.85
**Trained on the combined dataset and tested on the ISIC-2016 dataset**				
UNet	93.74	89.59	95.03	90.21
ResUNet	90.70	86.64	91.38	89.31
ResUNet++	92.71	90.02	94.34	92.19
**Trained on the combined dataset and tested on the ISIC-2017 dataset**				
UNet	83.74	80.65	85.00	85.15
ResUNet	79.83	77.89	81.33	83.37
ResUNet++	82.43	80.73	86.37	87.01

Table 9 presents the performance results of proposed methodology when trained on the combined dataset and tested on the combined testing dataset (ISIC-2016 and ISIC-2017), individual ISIC-2016 and ISIC-2017 datasets. ResUNet++ yielded the highest Jaccard index of 85.44% on the combined testing dataset. Similarly, when tested on individual testing datasets of ISIC-2016 and ISIC-2017, ResUNet++ produced the Jaccard Index of 90.02% and 80.73%, respectively, followed by UNet. The presented results prove the efficacy and robustness of the proposed methodology irrespective of the neural network architecture and the selection of the testing dataset, and the yielded results are in compliance with the inter-observer agreement of expert dermatologists. Figure 7 illustrates receiver operating curves (ROC) of the proposed skin lesion segmentation models.

#### Comparison with state-of-the-art

To further illustrate the robustness and effectiveness of the proposed methodology, we compared the results with state-of-the-art methods. As we trained the models on combined datasets, but for a fair comparison, we also evaluated our proposed methods by training the proposed models separately on ISIC-2016 and ISIC-2017 datasets. The trained models were tested on corresponding test images from both datasets. Our proposed UNet architecture achieved a Jaccard Index of 85.96% and 80.05% on ISIC-2016 and ISIC-2017 datasets, respectively. Table 10 presents the results of the proposed methodology on both datasets, it can be seen that the proposed method significantly outperformed the state-of-the-art frameworks on ISIC-2017 datasets while achieving a competitive Jaccard Index on the ISIC-2016 dataset.

**Table 10:** Comparison of proposed methodology with state-of-the-art techniques and competition participants.The results presented in this table are resulted from models trained and tested on individual ISIC-2016 and ISIC-2017 datasets.

Method	Dice	Jaccard	Precision	Recall
**Trained and tested on ISIC-2016**				
Team-EXB^13^	91.00	84.30	96.50	91.00
Team-CUMED^23^	89.70	82.90	91.10	95.70
Team-Rahman^13^	89.50	82.22	88.00	96.90
Bi et al.^5^	91.18	84.64	92.17	96.54
Yuan et al.^6^	91.20	84.70	96.60	91.80
Lesion Net^11^	92.39	**86.47**	96.45	93.62
*Proposed UNet*	90.39	85.96	87.46	92.76
*Proposed ResUNet*	88.33	84.15	84.75	92.22
*Proposed ResUNet++*	90.27	85.88	93.60	86.27
**Trained and tested on ISIC-2017**				
Yuan et el. (CDNN)^6^	84.90	76.50	97.50	82.50
Li et al.^24^	84.70	76.20	97.80	82.00
Bi et al. (ResNet)^25^	84.40	76.00	98.50	80.20
Lin et al. (UNet)^7^	77	62.00	–	–
Al-masni et al. (FrCN)^12^	87.08	77.11	96.69	85.40
Kashan et al. (ResUNet)^10^	85.80	77.20	–	–
Lesion Net^11^	87.87	78.28	96.08	86.23
*Proposed UNet*	90.39	**80.05**	86.77	82.17
*Proposed ResUNet*	79.28	77.66	82.46	80.47
*Proposed ResUNet++*	82.96	80.03	84.01	85.26

Significant values are in bold.

### Qualitative analysis

The complementary aim of study was to bridge the gap between research and clinical application of the skin lesion segmentation frameworks. Figure 6 show the qualitative results of the proposed segmentation methodology for ISIC-2016 and ISIC-2017 datasets. The proposed framework, irrespective of the utilized neural network model, predicts skin lesion masks precisely.

**Figure 8:**
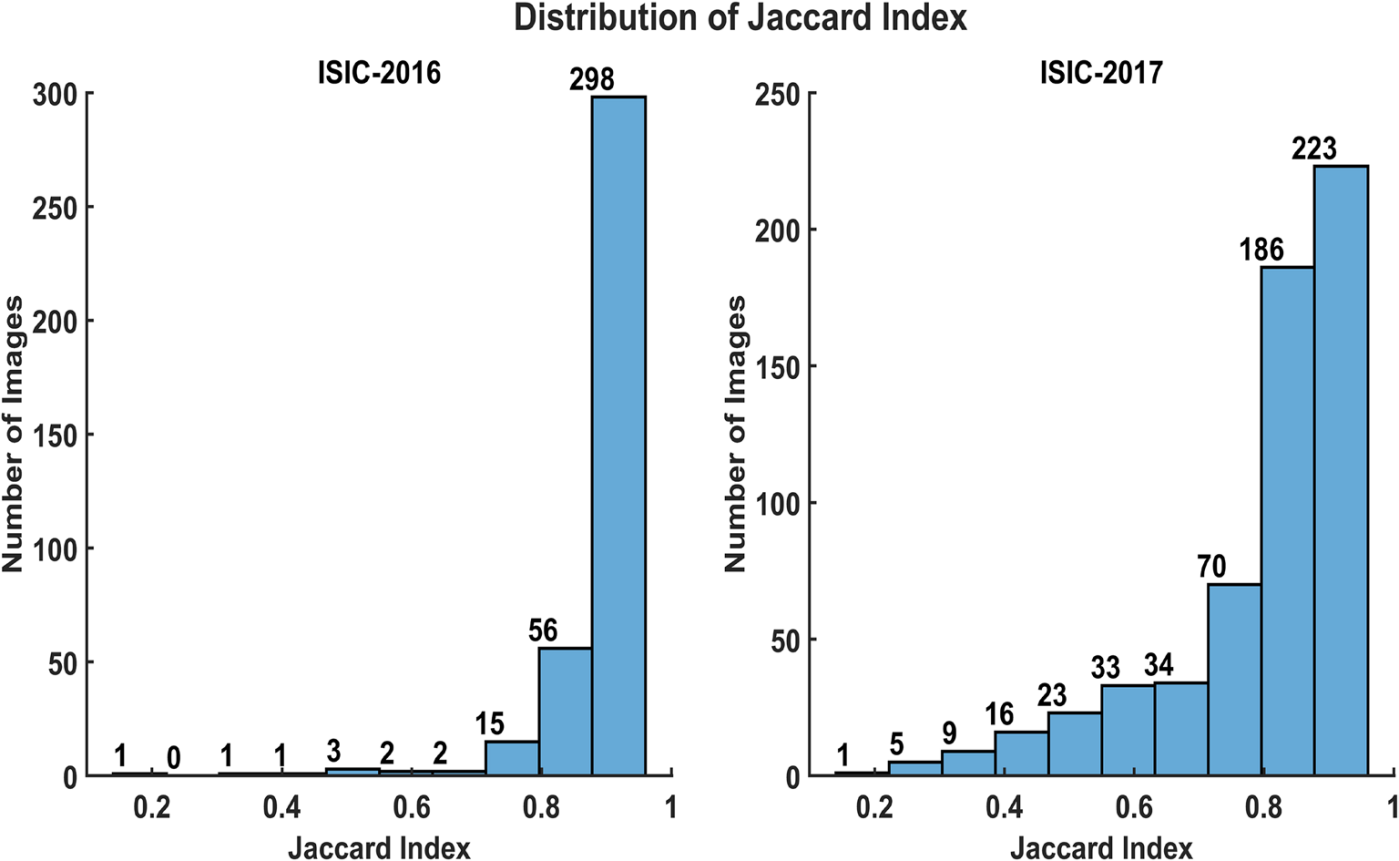
Distribution of jaccard index on ISIC-2016 and ISIC-2017 dataset. Each bar is labelled with number of images falling in the bin range of bars.

**Figure 9:**
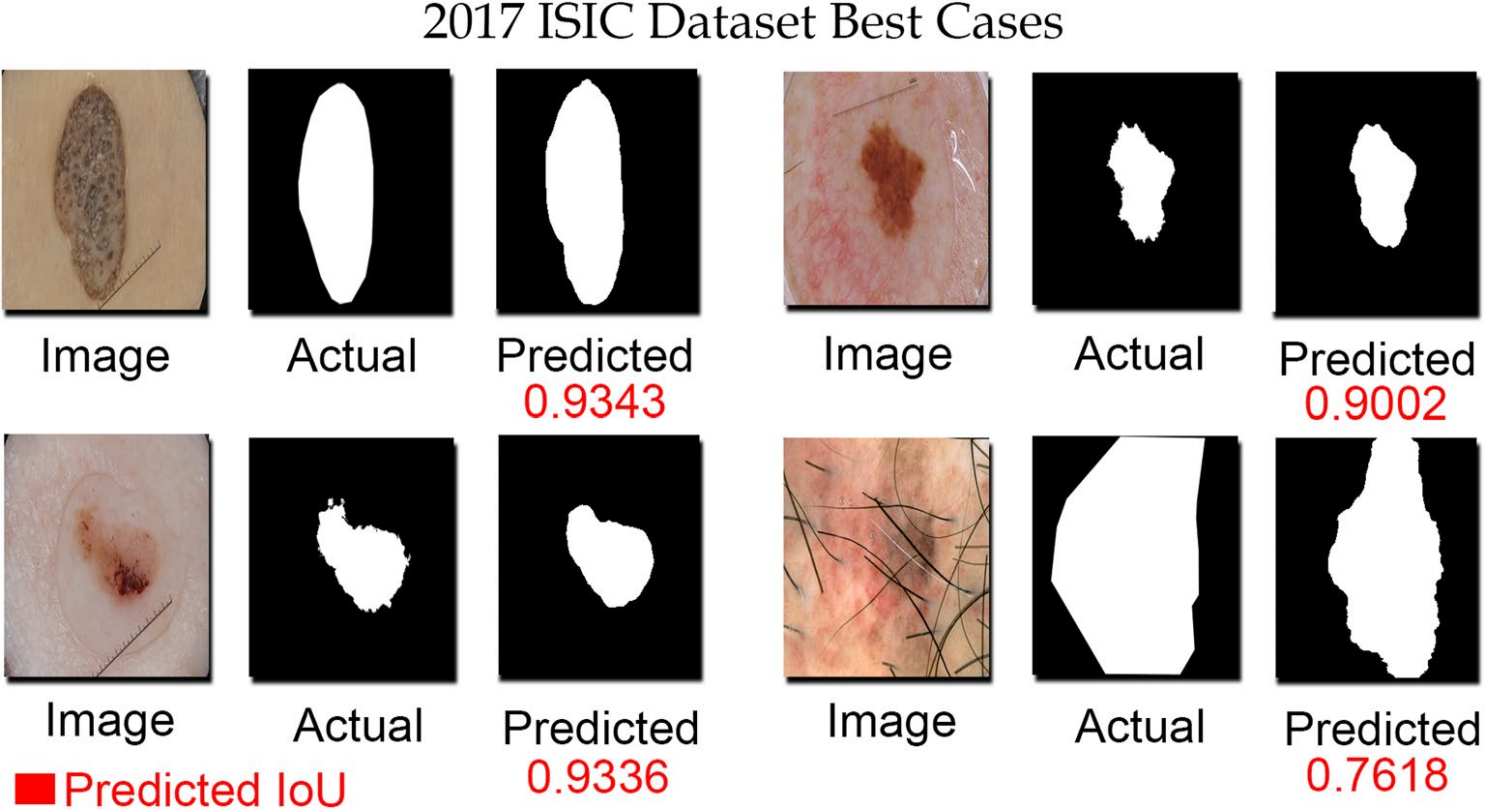
Some exemplary segmented images from ISIC-2017 dataset. The proposed skin lesion technique can handle the dermoscopic images acquired in different conditions. The model performed well on images with low contrast, invisible lesions, and images with hair structures.

**Figure 10:**
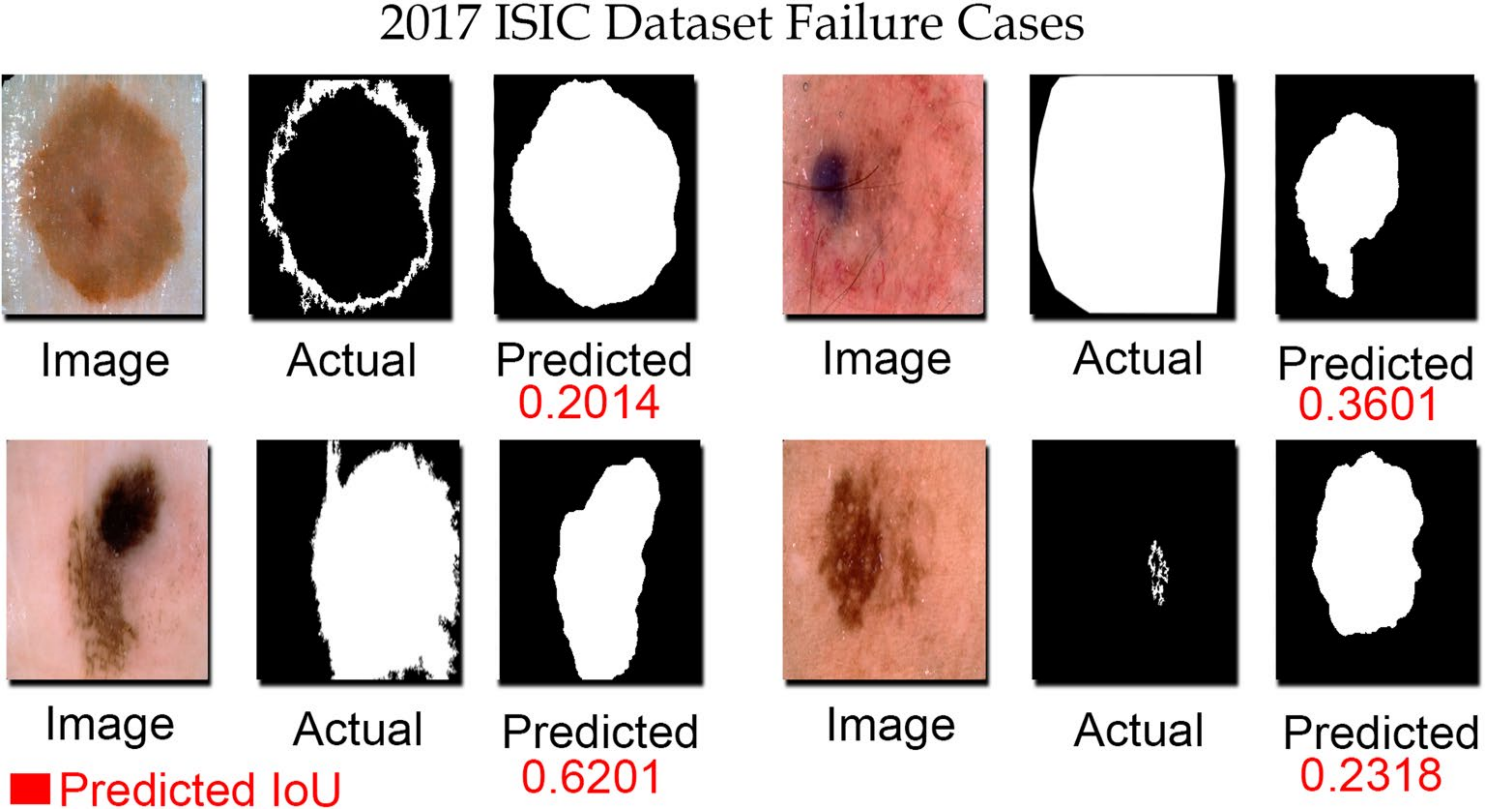
Failure cases in ISIC-2017 dataset. The provided annotated ground truth masks of some images are annotated incorrectly (subjective assessment). The model still predicted the masks quite resembled with the actual lesions, but the IoU score was low since it was assigned on the basis of provided versus predicted mask.

Figure 8 shows the distribution of jaccard index for ISIC-2016 and ISIC-2017 datasets. 93.40% of the predicted masks achieved a jaccard index above the inter-observer agreement of dermatologists for the ISIC-2016 dataset, which complies with subjective assessment of the skin lesion segmentation tasks. However, for the ISIC-2017 dataset 68.16% of the test images achieved a jaccard index of more than 80%, which is still higher than the top participant (57.83%) of the ISIC-2017 challenge^13^. The ISIC-2017 dataset contains testing images with greater variations in shape and size of the skin lesion also the poor contrast of skin lesion and background makes the segmentation task more challenging. Despite these challenges, our proposed framework detect and segment skin lesion precisely. Figure 9 shows some segmentation examples from the ISIC-2017 dataset, the proposed method can handle skin lesion images with varying conditions.

Even though our proposed methodology performed competitively relative to the previously reported accuracies on both datasets, there are some failure cases in the ISIC-2017 testing dataset. 20% of the testing images in the ISIC-2017 dataset achieved jaccard index below 70%, which are considered as failure cases. The reasons for such failure cases include: (1) low contrast of skin tumors such that the skin tumor is not distinguishable at all (2) lose ground truth annotations of skin lesions as few images in the testing dataset have masks that are not tight to skin lesion itself, and (3) annotation mistakes present in the dataset such as the presence of rulers and/or presence of skin tumor on borders of the images. Figure 10 illustrates some of the example images from the ISIC-2017 dataset, on which the jaccard index was lower than 70%. Upon examination, it has been found that the provided masks were annotated incorrectly. The predicted masks by proposed methodology quite resembled actual skin lesions presented in the dermoscopic images, however, the actual masks were annotated incorrectly leading to the lower jaccard index for such images.

## Discussion and conclusion

Instead of solely relying on the capabilities of on deep learning models, we incorporated a set of pre and postprocessing stages to handle the dermoscopic images acquired in different conditions with different characteristics. We utilized three different dense semantic segmentation architectures with optimized parameters along with pre and postprocessing stages. As a first step, we designed and proposed preprocessing algorithm to remove the unwanted objects and structures presented in the input images. The proposed preprocessing algorithm has shown significant improvement in overall performance of skin lesion segmentation, as illustrated in Fig. 4 and Table 5. The performance of the deep learning-based automatic diagnostic system dramatically also varies with sample size of the training set. Various studies have shown that the accuracy of skin lesion segmentation system increases with larger training dataset^5,6^. To increase the number of training set images, we utilized various image transformations. Upon investigating, we have identified the types of modification used in skin lesion segmentation tasks to increase the accuracy of the system. The specified combination of image transformations helped the proposed framework to learn better feature representations of skin lesion images.

We also conducted some experimentation to examine the effect of batch size and input image size. The results showed that the performance of the system increases with smaller batch size and higher input image size. All three proposed architectures showed a similar performance trend concerning the input image and batch size. To minimize the biasness, we investigated various loss functions to improve the accuracy of proposed system. By utilizing the identified combination of image transformations, batch size, image size, and loss function, we enhanced the performance and robustness of proposed skin lesion segmentation framework. As a postprocessing step, we incorporated TTA to maximize the accuracy of proposed system. Similar to train time augmentation, we generated various augmentations of the test images, which resulted in an increment in the performance of lesion segmentation system. To fine-tune the predicted masks, we extended the application of bayesian learning via CRF to enhance the qualitative and quantitative results of proposed methodology. Figure 6 and Table 9 illustrate the effect of proposed postprocessing methods on performance of skin lesion predictions.

To evaluate the performance of system, we measured various performance metrics; however, we only focused on jaccard index as per the suggestion of inter-observer agreement of expert dermatologists. Our proposed methodology resulted in a jaccard index of 84.60%, 81.07%, 85.44% with UNet, ResUNet, and ResUNet++ model when trained and tested on a combined dataset. The ResUNet++ model trained on the combined dataset yielded the highest jaccard index of 90.02% and 80.73% when tested on testing images of individual datasets of ISIC-2016 and ISIC-2017, respectively.

The proposed models were also trained and tested separately on ISIC-2016 and ISIC-2017 datasets to depict the efficacy of the proposed methodology. To the best of our knowledge, the current highest jaccard index of 86.47% and 78.28% (below than inter-observer agreement) for ISIC-2016 and ISIC-2017 was resulted by LesionNet^11^. The proposed methodology results are compared with the latest relevant literature and top participants of the competition and summarized in Table 10. On the ISIC-2017 dataset, our proposed UNet outperformed the previously reported highest Jaccard Index with a margin of 1.77%. Similarly, on the ISIC-2016 dataset, our proposed UNet achieved a similar Jaccard Index as of LesionNet. The complication involved in dermoscopic images makes the segmentation task more challenging, which can be observed in the case of the ISIC-2017 dataset. On the ISIC-2017 dataset, the recent state-of-the-art deep learning-based semantic segmentation techniques have failed to achieve the overall jaccard index in compliance with the inter-observer agreement, however, for the first time, our proposed method achieved the goal. Due to incorrect annotations of provided masks as shown in Fig. 10, in the testing dataset, the overall jaccard index could reach up to 80%.

It should be noted that both datasets achieved better segmentation results when tested using models trained on combined datasets in comparison to the models trained on the individual dataset. Although the combined dataset has only 150 more images than the ISIC-2017 dataset, it still improves the performance, validating that deep learning models provide better results with larger datasets. Although the proposed methodology outperformed previously reported accuracies, the proposed system has still some limitations. Firstly, the proposed method relies on the large training dataset, as it has been illustrated that the small dataset degrades the performance of the system. To mitigate this limitation, we combined both datasets and shown that the performance of the system increases significantly. Secondly, the performance is also affected by the different acquistion conditions of the captured images. Although, we have somehow limited the affect of different acquistion conditions by introducing TTA and CRF. In future, it is suggested that a guided protocol should be followed by practicing dermatology staff in clinical settings so that the captured dermoscopic image has a lesion at the center of the picture instead of borders with better contrast. It is also suggested that some other postprocessing techniques such as geodesic active contour levelset^26^ can be incorporated to mitigate the over and under segmentation failure cases.

This research proposed a detailed methodology for automated skin lesion segmentation incorporated with advanced pre and postprocessing techniques making the method highly scalable for dermoscopic images acquired in different retrieval environments. Unlike conventional deep learning-based semantic segmentation methods, the proposed methodology predicts a fine-tuned mask by employing bayesian learning, leading to the improvement in overall performance of lesion segmentation. To evaluate efficacy of the proposed methodology, we used ISIC-2016 and ISIC-2017 skin lesion datasets, and results showed that the proposed method outperformed the latest skin lesion segmentation approaches. To further increase the performance of system larger training datasets should be utilized to avoid under and over-segmentation cases. Due to the robustness and high scalability the proposed methodology can also be extended to other biomedical image segmentation challenges.

## Data Availability

The datasets generated during and/or analysed during the current study are available in the ISIC repository, https://www.isic-archive.com/#!/topWithHeader/wideContentTop/main.
